# The Telomeric Protein TRF2 Regulates Replication Origin Activity within Pericentromeric Heterochromatin

**DOI:** 10.3390/life11040267

**Published:** 2021-03-24

**Authors:** Serge Bauwens, Liudmyla Lototska, Stephane Koundrioukoff, Michelle Debatisse, Jing Ye, Eric Gilson, Aaron Mendez-Bermudez

**Affiliations:** 1 Faculty of Medicine Nice, Institute for Research on Cancer and Aging (IRCAN), CNRS, INSERM, Université Côte d’Azur, 06107 Nice, France; serge.bauwens@unice.fr (S.B.); l.lototska@imb-mainz.de (L.L.); 2 Institut Gustave Roussy, Sorbonne Université, UPMC University, 94805 Villejuif, France; stephane.koundrioukoff@gustaveroussy.fr (S.K.); michelle.debatisse@gustaveroussy.fr (M.D.); 3 International Laboratory in Hematology, Cancer and Aging, Pôle Sino-Français de Recherches en Sciences du Vivant et Génomique, Rui Jin Hospital, Shanghai Jiao Tong University School of Medicine, Shanghai 200025, China; yj11254@rjh.com.cn

**Keywords:** TRF2, heterochromatin, pericentromeric DNA, ORC, replication, telomeres

## Abstract

Heterochromatic regions render the replication process particularly difficult due to the high level of chromatin compaction and the presence of repeated DNA sequences. In humans, replication through pericentromeric heterochromatin requires the binding of a complex formed by the telomeric factor TRF2 and the helicase RTEL1 in order to relieve topological barriers blocking fork progression. Since TRF2 is known to bind the Origin Replication Complex (ORC), we hypothesized that this factor could also play a role at the replication origins (ORI) of these heterochromatin regions. By performing DNA combing analysis, we found that the ORI density is higher within pericentromeric satellite DNA repeats than within bulk genomic DNA and decreased upon TRF2 downregulation. Moreover, we showed that TRF2 and ORC2 interact in pericentromeric DNA, providing a mechanism by which TRF2 is involved in ORI activity. Altogether, our findings reveal an essential role for TRF2 in pericentromeric heterochromatin replication by regulating both replication initiation and elongation.

## 1. Introduction

Genomic DNA exhibits a higher-order nuclear organization that needs to be preserved to ensure the adequate physiological functioning of the cell. This compartmentalized nuclear structure is associated with DNA epigenetic modification and several associated proteins that are packed into two major types, euchromatin and heterochromatin [1]. In contrast to euchromatin, heterochromatin regions replicate late during the S phase and this timely process is important for the fine regulation of the cell cycle homeostasis.

Studies in fission and budding yeast have revealed that telomeric factors such as Rif1 and Taz1 ensure the firing of late replication origins throughout the genome [2]. In *Schizosaccharomyces pombe*, Taz1 delays the firing of several late-replication origins and recruits Ccq1 to assemble heterochromatin [3,4]. In human cells, the Taz1 orthologs, TRF1 and TRF2, have also replicative properties. In telomeres, TRF1 prevents ATR kinase signaling by recruiting the Bloom helicase (BLM) to facilitate lagging strand synthesis [5], while TRF2 prevents the accumulation of topological stress during replication by regulating the 5′exonuclease activity of Apollo [6] and by the recruitment of the BUB1-BUB3 complex, which in turn phosphorylates TRF1 [7]. In pericentromeric heterochromatin, TRF2 binds to satellite DNA II and III preferentially during the S phase and protects those regions against DNA damage response (DDR) activation by preventing fork stalling [8]. The recruitment of TRF2 to satellite DNA is not dependent on its sequence-specific telomeric binding (Myb) domain, but on its NH_2_-terminal domain, also known as the basic (B) domain, which has the capacity to recognize four-way DNA junctions in a sequence-independent manner [9,10]. A role of TRF2 during pericentromeric replication is to interact with the G4-resolving helicase RTEL1. The replicative function of TRF2 appears specific to telomeres and pericentromeres and not to other repetitive regions such as centromeres. Interestingly, this function is not limited to replication elongation but applies also to initiation. Indeed, in telomeres, TRF2 binds subunits of the origin recognition complex (ORC), such as ORC1 and ORC2, and regulates replication initiation [8,11,12,13]. In the Epstein–Barr virus (EBV) OriP, TRF2 and its partner, RAP1, bind to the telomeric DNA repeats present in EBV to ensure the recruitment of ORC and allow viral replication [14,15,16].

In this study, we explored the role of TRF2 in replication origin (ORI) density regulation in pericentromeric satellite DNA. We found that TRF2 is required for the association of ORC2 to this region. The ORI density within pericentromeric DNA was higher than within bulk genomic DNA and specifically controlled by TRF2.

## 2. Materials and Methods

The shTERF2 HeLa cell line was a gift from Lingner’s lab [17]. The cell line was grown in DMEM media supplemented with 10% fetal bovine serum tetracycline-free (Takara, Saint-Germain-en-Laye, France). The cultures were routinely tested for mycoplasma contamination.

### 2.1. Transient Transfections

siControl and siTRF1 (On-Target Plus SMARTpool, Dharmacon) RNA transfections were performed using the Dharmafect1 transfection reagent (Dharmacon, Lafayette, CO, USA) for 72 h, according to the manufacturer’s recommendations.

### 2.2. Real-Time qPCR

Gene expression for siRNA and rescue experiments was estimated by qPCR. Total RNA was obtained using the RNeasy mini kit (Qiagen, Hilden, Germany), while cDNA was generated using the High-Capacity RNA-to-cDNA kit (Fisher Scientific, Illkirch, France). qPCR was performed in triplicate using the Applied StepOne Plus system (Fisher Scientific, Illkirch, France) with SYBR green master mix (Roche, Basel, Switzerland).

### 2.3. Western Blotting

Protein extraction was obtained by incubating cell pellets in radio immunoprecipitation (RIPA) buffer at 4 °C. Proteins were separated by SDS-PAGE electrophoresis using commercial polyacrylamide gels (NuPAGE Mini gels, Fisher Scientific, Illkirch, France). Proteins were transferred to nitrocellulose membranes by a wet transfer system, followed by 1-h blocking with 1× PBST in 5% skimmed milk. Primary antibodies were incubated overnight at 4 °C, while secondary antibodies were incubated for 1 h at room temperature. Membranes were developed using the ECL detection system (Fisher Scientific, Illkirch, France) and exposed in the Fusion Solo apparatus (Vilbert Lourmat, Marne-la-Vallee, France). Antibodies used for Western blotting were the following: anti-TRF2 (Novus Biologicals, Littleton, CO, USA; NB110-57130), anti-TRF1 (Santa Cruz Biotechnology, Santa Cruz, CA, USA; sc-6165), anti-beta Actin (Abcam, Cambridge, UK, ab8227), anti-GAPDH (Novus Biologicals, Littleton, CO, USA, NB100-56875), anti-mouse HRP (Vector, Les Ulis, France, PI-2000), anti-rabbit HRP (Vector, Les Ulis, France, PI-1000).

### 2.4. DNA Combing

DNA combing was performed as described previously [8]. Briefly, HeLa cells were incubated with doxycycline for 5 days to induce shTRF2 expression. Before harvesting the cells, IdU was added to the media for 30 min (20 µM final concentration), followed by 30 min CldU (100 µM final concentration) and finally 1 mM thymidine for 5 min. Cells were trypsinized and embedded in 2% low melting agarose plugs followed by protein digestion with proteinase K. Next, agarose plugs were melted, and the DNA was stretched onto silanized coverslips at a constant speed (300 µm/s) and denatured in 1 N NaOH for 8 min, followed by ethanol dehydration. DNA was hybridized at 37 °C overnight with biotin-conjugated Peptide Nucleic Acids (PNA) probes (Sat III: Biotin-O-TTCCATTCCATTCCATTCCA; centromere: Biotin-O-AAACTAGACAGAAGCATT). Slides were incubated with the following antibodies: mouse anti-BrdU (BD Biosciences, San Jose, CA, USA, 347583), rat anti-BrdU (Abd Serotec, Oxford, UK, OBT0030), goat anti-mouse Alexa 488 (Fisher Scientific, Illkirch, France, A11029), goat anti-rat Alexa 555 Fisher Scientific, Illkirch, France, A21434), mouse anti-ssDNA (Millipore, Guyancourt, France, MAB3034), goat anti-mouse Cy5.5 (Abcam, Cambridge, UK, ab6947), donkey anti-goat Cy5.5 (Abcam, Cambridge, UK, ab6951), Streptavidin Alexa 488 (Fisher Scientific, Illkirch, France, S32354), rabbit anti-streptavidin biotin-conjugated (Rockland, 200-406-095), goat anti-mouse Cy5.5 (Abcam, Cambridge, UK, ab6947), goat anti-rat Alexa Fluor 555 (Fisher Scientific, Illkirch, France, A21434). All antibodies were incubated for 30 min at room temperature, with 1X PBS washes in between antibodies (except after incubation with primary anti BrdU antibodies, where washes were 6 min, at room temperature, in NaCl 0.5 M, Tris 20 mM pH 7.8, Tween20 0.5%).

Images were acquired with an epifluorescence microscope (Axio Imager Z2, Carl Zeiss, Jena, Germany) equipped with a 63× objective, a Cool-SNAP HQ2 camera (Roper Scientific Paray-vieille-poste, France) and MetaMorph software (Roper Scientific). To calculate inter-origin distance, images were analyzed with the ImageJ (FIJI) software. Replication tracks ending at the same point as the pericentromeric, centromeric or bulk DNA counterstained fibers were omitted.

### 2.5. Chromatin Immunoprecipitation (ChIP)

ChIP was carried out as shown before [8], with the following specifications. Each ChIP replicate was performed with 20 µg of formaldehyde crosslinked chromatin and 5 µg of the following antibodies: anti-ORC1 (Santa Cruz Biotechnology Santa Cruz, CA, USA; sc-23887), anti-ORC2 (Santa Cruz Biotechnology; sc-32734). The immunoprecipitated chromatin was de-crosslinked, phenol/chloroform-purified and ethanol-precipitated. Next, the DNA was denatured and spotted onto a N+ nylon membrane (Fisher Scientific, Illkirch, France) using a slot blot apparatus. The membrane was incubated with a radioactively labeled satellite III probe. The membranes were exposed to phosphorimager screens and the signal intensity quantified with ImageQuant software.

### 2.6. Statistics

Statistics and graphs were acquired using the GraphPad Prism v7 software. *p* values were obtained using non-parametric tests. Differences were considered statistically significant when the *p* value was < 0.05 (** *p* < 0.001; *** *p* < 0.0001).

## 3. Results

### 3.1. TRF2 Controlled ORC Association to Pericentromeric Satellite DNA

**Figure 1:**
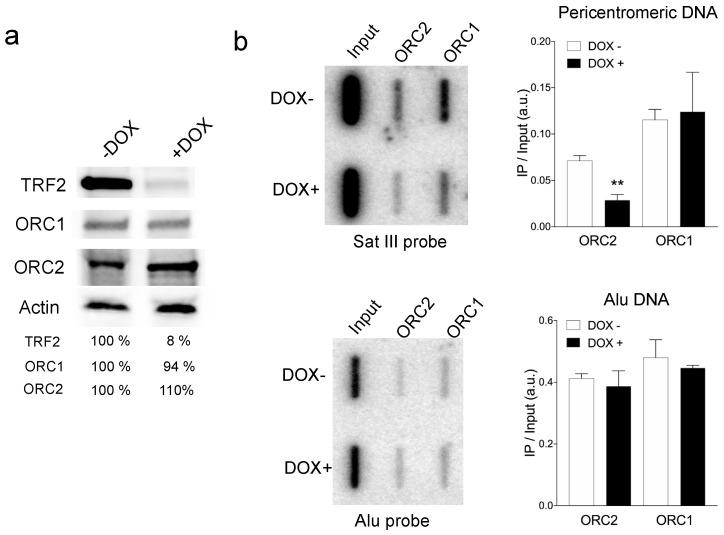
The ORC complex in pericentromeric regions. (**a**) Western blotting showing the expression of TRF2 in HeLa cells treated with doxycycline (DOX) for 5 days. The percentage of the remaining signal normalized to actin is shown at the bottom of the gel; (**b**) Slot blot showing the presence of ORC1 and ORC2 in pericentromeric regions. Membrane was hybridized with a radioactive satellite III DNA probe (left panel). Quantification of the signal obtained by normalizing the immunoprecipitated signal (IP) to that of the input is shown (right panel). Bars show SD of the mean of three biological replicates. (** *p* < 0.001; Mann–Whitney U test).

We asked whether TRF2 is able to recruit ORC to non-telomeric repeats, specifically in pericentromeric DNA. For this, we performed ChIP experiments of the ORC subunits ORC1 and ORC2 in an inducible HeLa cell line that expresses sh*TERF2* upon doxycycline addition for five days (Figure 1a and Supplementary Figure S1a). The immunoprecipitated DNA was spotted onto slot blots and hybridized with a radioactively labeled satellite III (Sat III) probe, which is one of the most representative repeats of pericentromeric DNA (Figure 1b and Supplementary Figure S1b). We found that the association of ORC2 to Sat III regions was significantly reduced after four days of TRF2 downregulation, while ORC1 was not affected. These results show that TRF2 is required for the recruitment of ORC in pericentromeric DNA.

### 3.2. TRF2 Regulates Origin Density in Pericentromeric Heterochromatin

**Figure 2:**
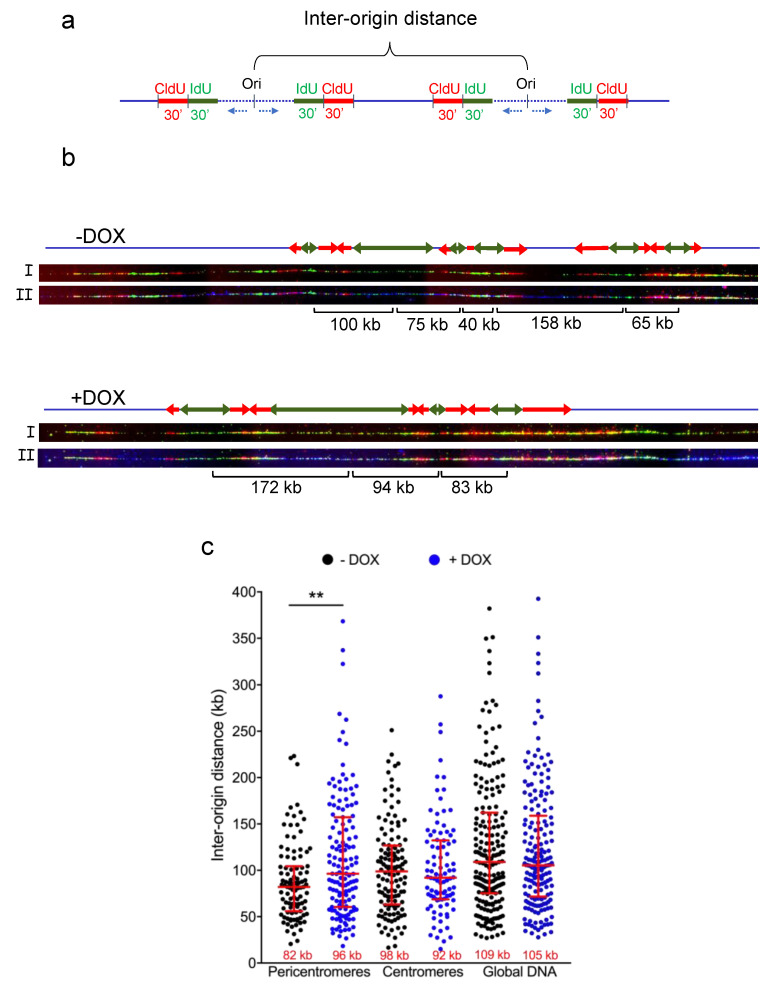
Origin of replication density in shTERF2-expressing HeLa cells. (**a**) Scheme showing the experimental setting used to measure the distance between two origins of replication (inter-origin distance; IOD); (**b**) Representative images of DNA combing at pericentromeres showing only the signal of IdU (green), IdU (red) top image (I) or with satellite III PNA probe signal (blue) bottom image (II). Green and red arrows indicate IdU and CldU labeling, respectively. IOD distance is shown below the images; (**c**) Quantification of IOD expressed in kilobases (kb) for HeLa cells expressing a doxycycline (DOX)-inducible shTERF2 system. Median length is shown at the bottom of the scatter plots. Bars represent the median +/− interquartile range (** *p* < 0.001; Mann–Whitney U test).

In order to study the role of TRF2 in replication initiation in pericentromeres, we set out to examine the ORI density in pericentrolmeric heterochromatin in cells containing a doyxycline-inducible shTERF2 construct. After 5 days of doxycycline exposure, we added IdU, followed by CldU for 30 min (Figure 2a), and we stretched the DNA onto treated slides using the DNA combing technique [18]. We combined DNA combing experiments with Sat III PNA probe hybridization to directly analyze origin density in pericentromeric, centromeric and bulk DNA by measuring the distance between two contiguous origins of replication (Figure 2b,c). We found that the ORI density is higher within pericentromeric DNA as compared to that of centromere and global DNA, more likely balancing the slower replication speed found in heterochromatin (82 kb at pericentromeric DNA compared to 109 in bulk DNA) [8,19]. Upon TRF2 downregulation, the ORI density decreased within pericentromeric DNA (from 82 kilobases [kb] to 96 kb) but not within centromeric DNA or bulk DNA. We also downregulated the expression of TRF1 (Supplementary Figure S2a) and did not find any alteration in origin density at those sites (Supplementary Figure S2b).

### 3.3. The B Domain of TRF2 Is Required for Origin Density Regulation

**Figure 3:**
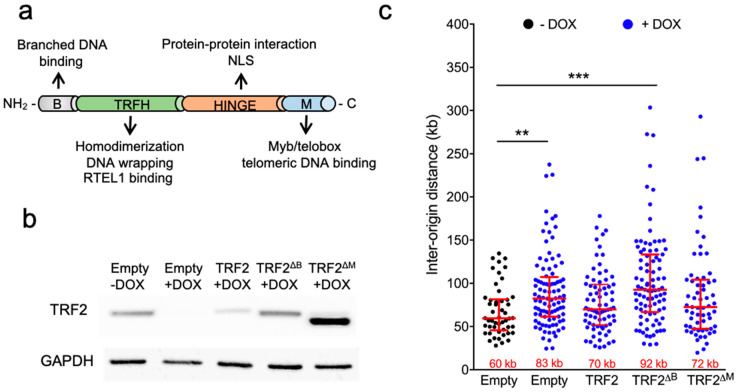
Effect of TRF2^ΔB^ and TRF2^ΔM^ on origin density. (**a**) Relevant TRF2 protein domains and functions; (**b**) TRF2 expression of HeLa cells treated for 5 days with doxycycline (DOX). One day after DOX was added to the media, the indicated lentivirus-expressing plasmids were added to the culture; (**c**) Quantification of inter-origin distances at pericentromeric regions expressed in kilobases (kb) for the conditions described in *b*. Median length is displayed at the bottom of the scatter plots. Bars represent the median +/− interquartile range (** *p* < 0.001; *** *p* < 0.0001; Mann–Whitney U test).

To explore which domain of TRF2 is involved in the control of ORI density, we transduced the shTERF2-inducible HeLa cell line with lentiviral vectors expressing either the full-length TRF2 protein or two truncated forms, one lacking the B domain and the other lacking the telobox Myb telomeric DNA binding domain of TRF2, named the M domain (Figure 3a). We inhibited the expression of the endogenous TRF2 protein by the addition of doxycycline; on the next day, the cells were transduced for four days with the corresponding lentivirus, and DNA combing at pericentromeric DNA was performed (Figure 3b). The expression of the full-length TRF2 and its truncated form, TRF2^ΔM^, rescued ORI density; however, TRF2^ΔB^ did not restore it. These results show that the B domain, but not the telobox domain, of TRF2 is required for ORI density regulation in pericentromeres.

## 4. Discussion

The main result of this work was to reveal the specific role of TRF2 in replication initiation at pericentromeric regions. First, we found that the recruitment of ORC2 in pericentromeric DNA is dependent upon TRF2. This result is in agreement with the previously reported interaction between TRF2 and ORC2 [13,15,16]. Since ORC2 binds preferentially to open chromatin regions [20], highly condensed chromatin regions, such as pericentromeres, might need specific factors, such as TRF2, to facilitate its recruitment. In further agreement with this view, in telomeres, TRF2 also recruits ORC [21]. ORC is also linked to the formation of heterochromatin in yeast [22] and mammalian cells [23], where the ORC-associated protein ORCA/LRWD1 stabilizes ORC binding in repressive chromatin and helps with chromatin organization. Thus, it is tempting to hypothesize that TRF2, through the recruitment of ORC in pericentromeres, also contributes to the establishment and/or the maintenance of the heterochromatin state.

We also found that the inter-ORI distance increased upon TRF2 downregulation in pericentromeres but not in centromeres or bulk genomic DNA. This inter-ORI effect is not observed upon TRF1 inhibition. This result is counterintuitive considering that TRF2 downregulation triggers DDR and fork stalling in pericentromeres [8], two events expected to decrease the inter-ORI distance [19]. Thus, TRF2 appears to play a specific role in regulating pericentromeric ORI activity dissociated from its function in pericentromeric replication elongation. This dual replicative function of TRF2 is expected to act synergistically to profoundly perturb pericentromeric replication and stability upon TRF2 downregulation [8].

Mechanistically, the role of TRF2 in replication initiation in pericentromeres is likely to involve its interaction with ORC2, since we showed that ORC2 requires TRF2 to bind pericentromeric DNA and that the inter-ORI distance depends upon the basic N-terminal TRF2 domain, known to interact with ORC2 [8]. Accordingly, the expression of the TRF2^ΔB^ mutant form reduces ORI formation in telomeres by disrupting ORC and the replicative helicase MCM3 [21]. Other properties of TRF2 could also be involved in its role in pericentromeric replication initiation since it was reported that it associates to ORCA/LRWD1 [24] and that, in addition to the basic N-terminal domain, the TRF2 dimerization domain is required for ORC recruitment [25]. An interesting possibility would be that the pericentromeric ORI activity also relies on the ability of TRF2 to bind and unwind the formation of a G-quadruplex structure within the G-rich satellite DNA repeats [26].

A limitation of this study is not to have analyzed the role of TRF2 in replication initiation in normal cells, particularly because it was reported that immortalization alters the ORI distribution within heterochromatin [26]. Nevertheless, besides having revealed a specific role of TRF2 in ORI activity within pericentromeres, this study paves the way towards a better understanding of the specific determinants of replication initiation within the highly repeated heterochromatin regions, a process which is still poorly investigated.

## Supplementary Materials

The following are available online at https://www.mdpi.com/2075-1729/11/4/267/s1, Figure S1: TRF2 is important in ORC recruitment pericentromeric DNA. Figure S2: TRF1 is not involved in origin density regulation.
