# Supplementary material for: The Telomeric Protein TRF2 Regulates Replication Origin Activity within Pericentromeric Heterochromatin
Source: Life (Basel). 2021 Mar 24;11(4):267. doi: 10.3390/life11040267 (PMC8063955; doi:10.3390/life11040267)
Supplement: Supplementary file 1 [file life-11-00267-s001.pdf]

The Telomeric Protein TRF2 Regulates Replication Origin Activity within Pericentromeric Heterochromatin

Supplementary Materials:

Figure S1. TRF2 is important in ORC recruitment at pericentromeric DNA.

Figure S2. TRF1 is not involved in origin density regulation.

Figure S1. TRF2 is important in ORC recruitment at pericentromeric DNA.

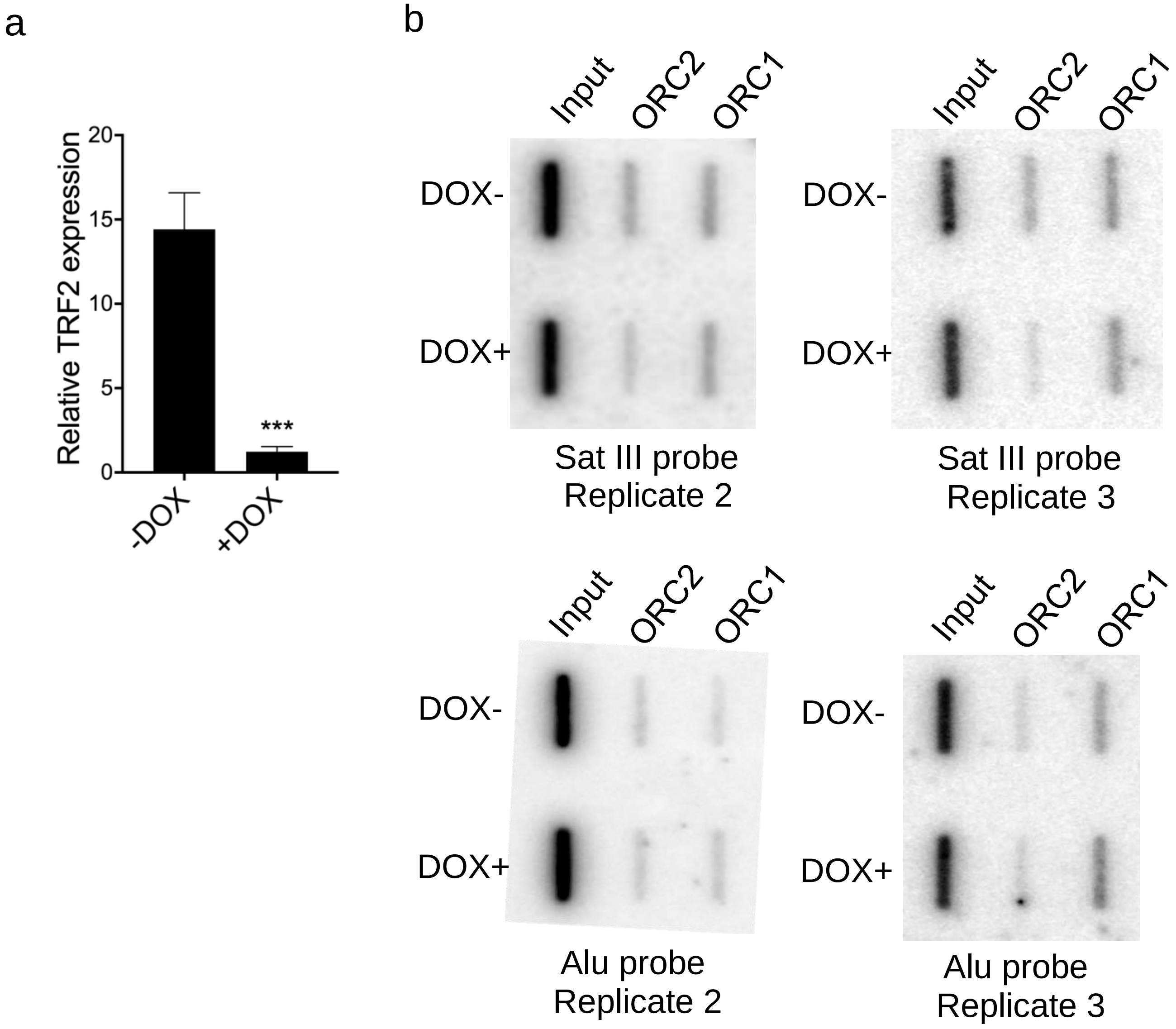

(**a**) qPCR of HeLa cells incubated for 5 days with doxycycline to inhibit the expression of TRF2. Error bars represent the mean ± SD of three independent experiments. Statistical analyses were performed using two-tailed Student's *t*-test (****P* < 0.0001). (**b**) Second and third replicate of the ChIP experiment show in Figure 1b.

Figure S2. TRF1 is not involved in origin density regulation.

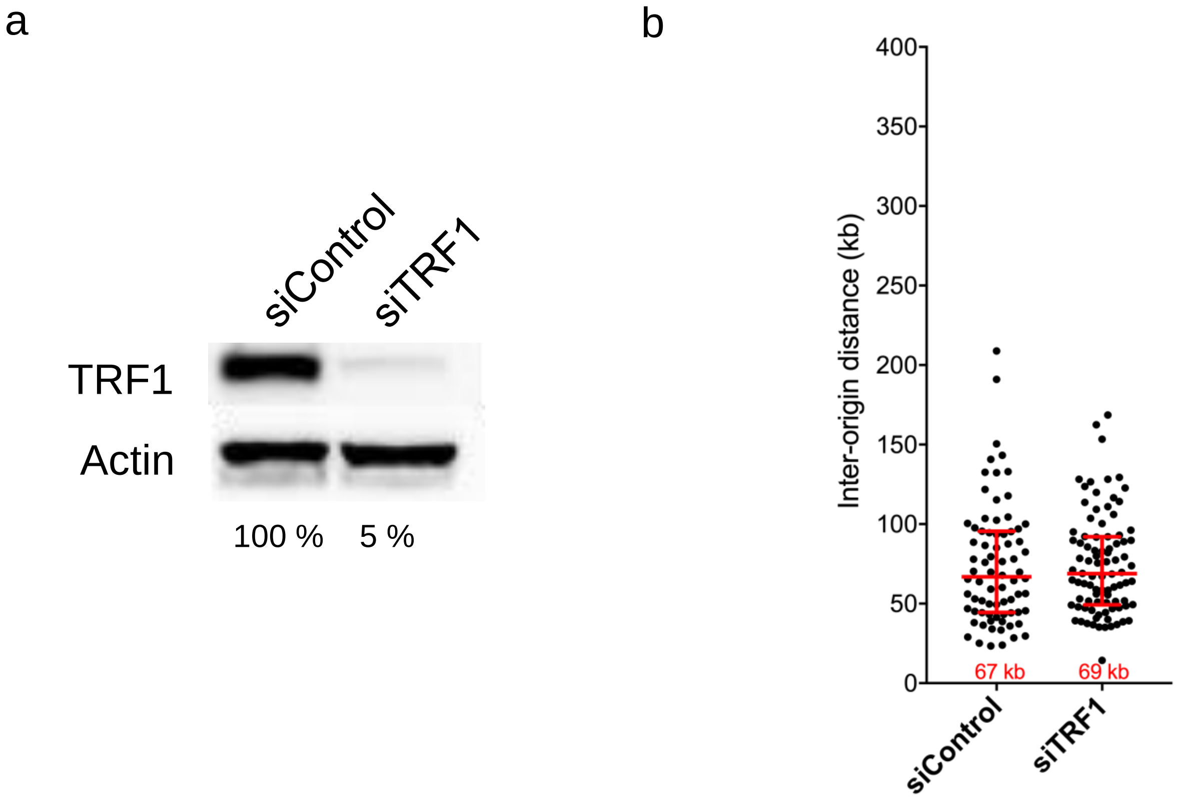

(**a**) Western blotting of HeLa cells treated for three days with the corresponding siRNA. The percentage of the signal of TRF1 in relationship to actin is shown at the bottom of the gel. (**b**) Scatter plot showing the distribution of the distance Between 2 origins of replication. The median +/- interquartile range is shown.
